# Supplementary material for: Chromothripsis during telomere crisis is independent of NHEJ, and consistent with a replicative origin
Source: Genome Res. 2019 May;29(5):737–49. doi: 10.1101/gr.240705.118 (PMC6499312; doi:10.1101/gr.240705.118)
Supplement: Supplemental Material [file supp_gr.240705.118_Supplemental_file_1.zip › contigs/annotated_contigs/DB54/contig.2.DB54_length_312_mean_cov_4.80769230769.docx]

**DB54_length_312_mean_cov_4.80769230769**

```
CACTAAAGGGACCCTGGTATTAAGCCTCCTTAATGCAAGAAAATCATCTATCCCTGGTCTTTATTTTAGCTGTTGTTTGAAATACATGT
 >chr10:124153015-124153291 - E=2e-155
TACAAAAAAAGTATTTCTTATTTTTGGGACAGCTTCAATTGTTATTTTTTTCTGTGACTCAAGTTCTAATTTTCTAAAACCTAATTTGC

CACCAGAGAGAGTATTTCTCTCTCGAGGGAAAAATATATACAGAAGCATTAATTTTTATCAAATAAACTTAGTTTCACCTTCTGAAATT

CAAACAAAA|AAACC|GGCATGTCTTAGACAGGGGGGT|GAGGCACCC               >chr10:124028901-124028923 - E
```
